# Assessing coastal wetland vulnerability to sea-level rise along the northern Gulf of Mexico coast: Gaps and opportunities for developing a coordinated regional sampling network

**DOI:** 10.1371/journal.pone.0183431

**Published:** 2017-09-13

**Authors:** Michael J. Osland, Kereen T. Griffith, Jack C. Larriviere, Laura C. Feher, Donald R. Cahoon, Nicholas M. Enwright, David A. Oster, John M. Tirpak, Mark S. Woodrey, Renee C. Collini, Joseph J. Baustian, Joshua L. Breithaupt, Julia A. Cherry, Jeremy R. Conrad, Nicole Cormier, Carlos A. Coronado-Molina, Joseph F. Donoghue, Sean A. Graham, Jennifer W. Harper, Mark W. Hester, Rebecca J. Howard, Ken W. Krauss, Daniel E. Kroes, Robert R. Lane, Karen L. McKee, Irving A. Mendelssohn, Beth A. Middleton, Jena A. Moon, Sarai C. Piazza, Nicole M. Rankin, Fred H. Sklar, Greg D. Steyer, Kathleen M. Swanson, Christopher M. Swarzenski, William C. Vervaeke, Jonathan M. Willis, K. Van Wilson

**Affiliations:** 1 U.S. Geological Survey, Lafayette, Louisiana, United States of America; 2 Griffith Consulting Services at U.S. Geological Survey, Lafayette, Louisiana, United States of America; 3 Five Rivers Services, Lafayette, Louisiana, United States of America; 4 U.S. Geological Survey, Patuxent, Maryland, United States of America; 5 U.S. Fish and Wildlife Service, Lafayette, Louisiana, United States of America; 6 Grand Bay National Estuarine Research Reserve, Moss Point, Mississippi, United States of America; 7 Northern Gulf of Mexico Sentinel Site Cooperative, Dauphin Island, Alabama, United States of America; 8 The Nature Conservancy, Baton Rouge, Louisiana, United States of America; 9 University of South Florida, St. Petersburg, Florida, United States of America; 10 University of Alabama, Tuscaloosa, Alabama, United States of America; 11 U.S. Fish and Wildlife Service, Sanibel, Florida, United States of America; 12 South Florida Water Management District, West Palm Beach, Florida, United States of America; 13 University of Central Florida, Orlando, Florida, United States of America; 14 Nicholls State University, Thibodaux, Louisiana, United States of America; 15 Apalachicola National Estuarine Research Reserve, Eastpoint, Florida, United States of America; 16 University of Louisiana at Lafayette, Lafayette, Louisiana, United States of America; 17 U.S. Geological Survey, Baton Rouge, Louisiana, United States of America; 18 Louisiana State University, Baton Rouge, Louisiana, United States of America; 19 U.S. Fish and Wildlife Service, Winnie, Texas, United States of America; 20 U.S. Fish and Wildlife Service, Awendaw, South Carolina, United States of America; 21 Mission-Aransas National Estuarine Research Reserve, Port Aransas, Texas, United States of America; 22 U.S. Geological Survey, Jackson, Mississippi, United States of America; University of Sydney, AUSTRALIA

## Abstract

Coastal wetland responses to sea-level rise are greatly influenced by biogeomorphic processes that affect wetland surface elevation. Small changes in elevation relative to sea level can lead to comparatively large changes in ecosystem structure, function, and stability. The surface elevation table-marker horizon (SET-MH) approach is being used globally to quantify the relative contributions of processes affecting wetland elevation change. Historically, SET-MH measurements have been obtained at local scales to address site-specific research questions. However, in the face of accelerated sea-level rise, there is an increasing need for elevation change network data that can be incorporated into regional ecological models and vulnerability assessments. In particular, there is a need for long-term, high-temporal resolution data that are strategically distributed across ecologically-relevant abiotic gradients. Here, we quantify the distribution of SET-MH stations along the northern Gulf of Mexico coast (USA) across political boundaries (states), wetland habitats, and ecologically-relevant abiotic gradients (i.e., gradients in temperature, precipitation, elevation, and relative sea-level rise). Our analyses identify areas with high SET-MH station densities as well as areas with notable gaps. Salt marshes, intermediate elevations, and colder areas with high rainfall have a high number of stations, while salt flat ecosystems, certain elevation zones, the mangrove-marsh ecotone, and hypersaline coastal areas with low rainfall have fewer stations. Due to rapid rates of wetland loss and relative sea-level rise, the state of Louisiana has the most extensive SET-MH station network in the region, and we provide several recent examples where data from Louisiana’s network have been used to assess and compare wetland vulnerability to sea-level rise. Our findings represent the first attempt to examine spatial gaps in SET-MH coverage across abiotic gradients. Our analyses can be used to transform a broadly disseminated and unplanned collection of SET-MH stations into a coordinated and strategic regional network. This regional network would provide data for predicting and preparing for the responses of coastal wetlands to accelerated sea-level rise and other aspects of global change.

## Introduction

Due to their position at the land-sea interface, coastal wetlands are highly vulnerable to climate and land use change. Accelerated sea-level rise, coastal development, and other aspects of global change are expected to have a tremendous impact on coastal wetlands in the coming century [1–5]. In addition to providing fish and wildlife habitat, coastal wetlands protect coastlines, supply seafood, filter contaminants, store carbon, improve water quality, and provide recreational opportunities [6–9]. To protect these ecosystem goods and services, coastal scientists and environmental managers are increasingly challenged to better anticipate and prepare for the ecological effects of future change on coastal wetlands.

Coastal wetland responses to climate change, land use change, and accelerated sea-level rise will be modulated by biogeomorphic processes that affect wetland surface elevation [10–16]. Inundation and salinity regimes are tremendously important abiotic drivers that greatly influence the structure and function of coastal wetlands [17–19]. Small changes in wetland surface elevation relative to sea level can alter these important abiotic regimes, modify biogeomorphic processes, prompt ecological regime shifts, and, in the most extreme cases, lead to wetland loss via submergence and conversion to open water. Thus, an increasing need exists for long-term and high-temporal resolution surface elevation change data that can be incorporated into future-focused regional coastal wetland vulnerability assessments and predictive ecological models [11–13, 16, 20–23].

The surface elevation table (SET)-marker horizon (MH) approach (SET-MH, together) is a method for quantifying net wetland surface elevation change while accounting for various biological, geological, and hydrological processes that can occur within different segments of the soil profile (e.g., deep, shallow subsurface, and surface soil depths) [11, 20, 24–28]. Along with elevation change, sediment accretion, shallow subsidence (i.e., autocompaction), biotic contributions to root zone expansion, soil shrink-swell, bioturbation, and disturbance-induced peat collapse are all processes that have been measured using SET-MH methods, often in coordination with complementary process-focused measurements [12, 29–37]. Following up on the foundational work of Schoot and de Jong [38], van Eerdt [39], and Boumans and Day [40], the modern design, infrastructure, and terminology associated with the SET-MH approach was introduced by Cahoon et al. [24, 26, 31] and permits examination of the relative contributions of the various processes regulating surface elevation [11, 20, 25, 28].

The SET portion of the SET-MH approach entails the use of a portable lightweight mechanical leveling device (the SET) with movable fiberglass or metal pins that are lowered to the ground and used for high precision measurements of wetland surface elevation relative to a fixed benchmark. The SET is attached to benchmarks that represent different sections of the soil profile. Deep benchmarks are driven to refusal and capture elevation change across the entire depth of the soil profile [28]. In contrast, shallow benchmarks may be installed to the bottom of the active root zone to represent changes in elevation caused by root zone expansion or contraction. The confidence interval for elevation measurements made with SET [±1.3 mm; 24] is typically better than for other surface elevation measurement methods [20]. The MH portion of the SET-MH approach refers to the use of marker horizons to measure vertical accretion. An artificial marker horizon (e.g., a layer of feldspar or sand) is placed upon the soil surface, and material accumulation (composed of organic matter and mineral sediment) above this marker horizon is measured by taking soil cores. Both the SET and MH measurements can be measured repeatedly to track changes in elevation over time, and when examined together, they permit quantification of the relative contributions of different processes (e.g., shallow subsidence, surface accretion, root zone expansion) to net surface elevation change.

SET-MH technology has been shared globally so that data from different locations can be compared. The method has been adopted and used by scientists working in coastal wetlands in 32 countries across the world [11, 13, 16, 20–22, 25, 41]. However, high-vertical resolution surface elevation change measurements with SET-MH have historically been concentrated in certain regions and have often been employed at local scales to address specific research questions. Within the context of climate change, and accelerated sea-level rise in particular, there is an increasing need for elevation change networks that can inform regional coastal wetland vulnerability assessments [11, 12, 20–23]. From a modeling and monitoring perspective, there is an additional need for SET-MH stations that contain long-term data and are strategically distributed across ecologically-relevant abiotic gradients at both local and regional scales [13, 16]. Adequate distribution of SET-MH stations across ecologically-relevant abiotic gradients (e.g., gradients in relative sea-level rise, elevation, temperature, and precipitation) and time scales of change would provide critical data to improve model predictions of wetland vulnerability to sea-level rise. SET-MH stations have occasionally been installed across local abiotic gradients within an individual wetland complex; however, regional abiotic gradients have historically not been a primary consideration due in part to a lack of coordination between scientists and the absence of information regarding the actual distribution of SET-MH stations.

In this study, we investigated the following questions for the U.S. Gulf of Mexico region: (1) What is the distribution of wetland surface elevation monitoring infrastructure consisting of SET-MH stations?; (2) Within each of the five U.S. gulf coast states (i.e., Florida, Alabama, Mississippi, Louisiana, and Texas), when have SET-MH stations been installed and how comprehensive are the elevation records?; (3) What is the distribution of SET-MH stations within the dominant coastal wetland ecosystems (i.e., freshwater marsh, freshwater forest, salt marsh, mangrove forest, tidal flat, and subtidal ecosystems)?; and (4) What is the distribution of SET-MH stations across ecologically-relevant abiotic gradients (e.g., gradients in relative sea-level rise, elevation, temperature, and precipitation)?

## Materials and methods

### Study area and inventory of SET-MH stations

**Fig 1:**
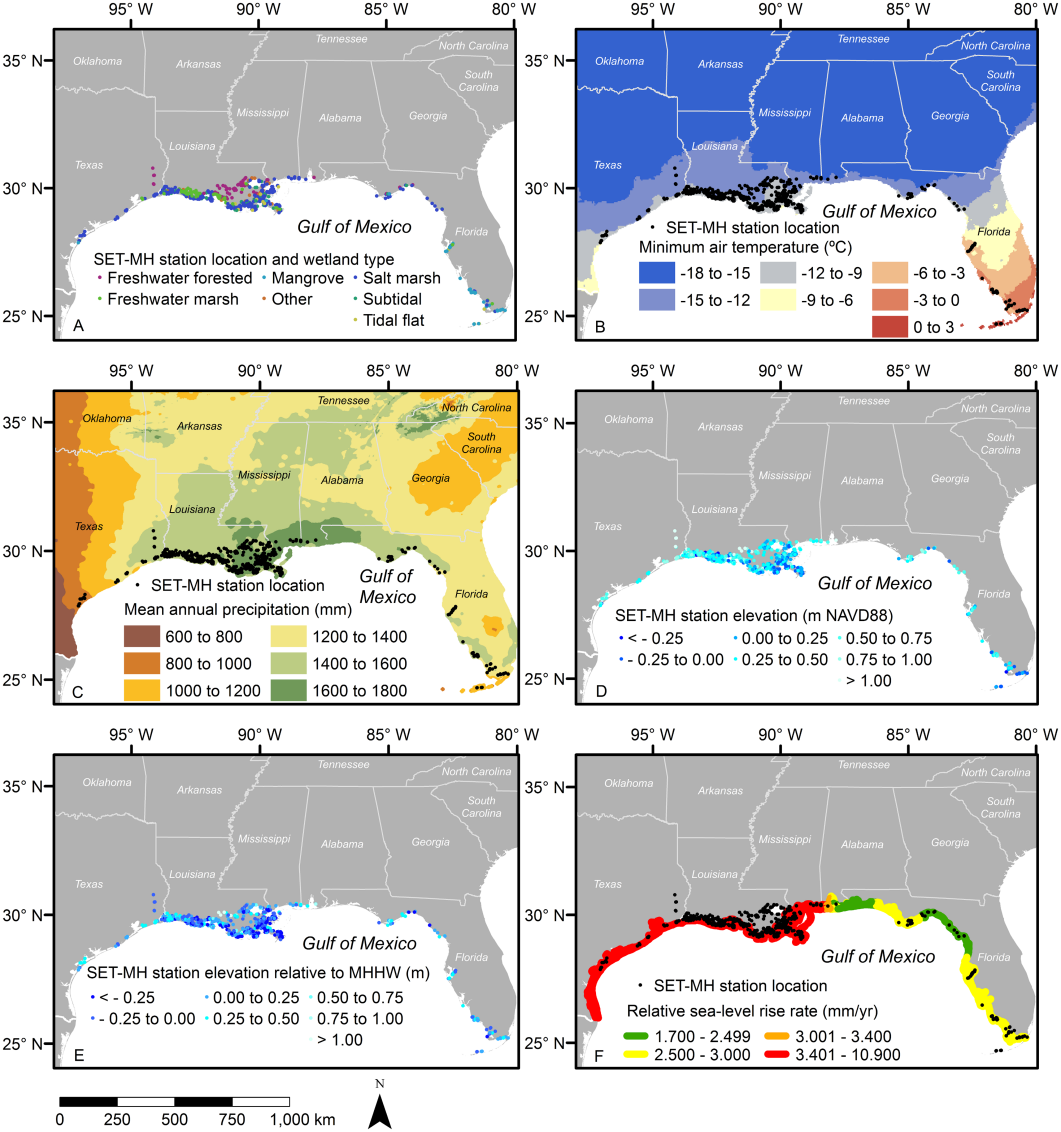
Maps of the distribution of coastal wetland surface elevation change infrastructure. Maps show the distribution of surface elevation table-marker horizon (SET-MH) stations along the U.S. Gulf of Mexico coast across: (A) wetland types, (B) minimum air temperature, (C) mean annual precipitation, (D) elevation, (E) elevation relative to mean higher high water (MHHW), and (F) rate of relative sea-level rise.

The study area included the coasts of all five U.S. states along the northern Gulf of Mexico (i.e., Florida, Alabama, Mississippi, Louisiana, and Texas) (Fig 1). We contacted federal, state, and university-affiliated scientists working with SET-MH data within this area to obtain the geographic coordinates and the installation year for each SET-MH station. See Lynch et al. [28] for a definition of an SET-MH station as well as a discussion of the distinctions between sample spaces, sample sites, and sample stations. Please note that while our inventory is extensive and includes most of the SET-MH stations in the region, our inventory is not fully exhaustive; in other words, it is possible that some stations in the region are not contained within this inventory. We used the SET-MH coordinates in combination with geospatial data to develop a dataset that included coastal wetland type, climate, elevation, tidal datum, and relative sea-level rise information for each SET-MH station. The SET-MH stations in our dataset include original SET, deep rod SET (RSET), and shallow RSET benchmarks [24, 26].

### Coastal wetland type data

The coastal wetland classification data associated with each SET-MH station location was obtained from the U.S. Fish and Wildlife Service (USFWS) National Wetland Inventory (NWI) [42]. Each SET-MH station was assigned one of the following wetland habitat types (NWI codes are in parentheses), as defined by NWI: salt marsh (E2EM), mangrove forest (E2FO or E2SS), tidal flat (E2US), subtidal habitat (codes that begin with E1), freshwater marsh (PEM), freshwater forested/scrub-shrub (hereafter referred to as freshwater forested) wetland (PFO or PSS), or other habitat (all other codes).

### Climate data

For each SET-MH station, we obtained 2.5-arcminute resolution climate data from the PRISM Climate Group (Oregon State University; http://prism.oregonstate.edu) [43]. These climate data include spatially-explicit estimates for mean annual precipitation and minimum air temperature (i.e., the absolute coldest temperature recorded) from 1981 to 2010. See Osland et al. [44–47] for descriptions of the importance of these climatic variables.

### Elevation, tidal datum, and relative sea-level rise data

We estimated the surface-level elevation relative to an orthometric datum (North American Vertical Datum 1988; NAVD88) for each SET-MH station using digital elevation models (DEM) developed from airborne light detection and ranging (lidar) data. We would have preferred to have in-situ elevation observations via global navigation satellite system and real-time kinematic methods for each SET-MH station, as lidar elevation uncertainty in densely vegetated areas such as marsh can be high [48–51]; however, such data were not consistently available. Nevertheless, these lidar-derived data are useful for providing an initial region-scale evaluation of the distribution of SET-MH stations across elevation gradients. For Florida, we used a 15-m horizontal resolution bare-earth DEM created by the University of Florida GeoPlan Center. For Louisiana, we used a 15-m resolution DEM developed from the USGS 3D Elevation Program Coastal National Elevation Dataset topobathymetric model of the northern Gulf of Mexico. For Mississippi and Texas, we utilized 10-m resolution DEMs created by the National Oceanic and Atmospheric Administration (NOAA) [52]. For Alabama, we used 5-m resolution DEMs created by NOAA [52]. In addition to the orthometric datum, we also estimated the vertical position of each SET-MH station relative to a local tidal datum elevation [mean higher high water (MHHW)] [3], using VDatum 3.1 [53]. We would have preferred to use tidal datum data derived from local gages adjacent to each SET-MH station; however, such data were not readily available. VDatum transformation estimates are modeled and also contribute to vertical uncertainty [54]. For the VDatum regions in this study, the mean VDatum-associated uncertainty is about 9.8 cm with a standard deviation of 4.4 cm. We used data from the USGS Coastal Vulnerability Index [55] to coarsely deduce relative sea-level rise rates for each SET-MH station.

In addition to the region-wide assessment of the distribution of SET-MH stations across elevation gradients, we also evaluated the elevation-based distribution of SET-MH stations within each of the following nine tidal datum regions defined in VDatum: (1) FLsouth (south Florida); (2) FLwest (central Florida); (3) FLapalach (north Florida); (4) FLpensac (Pensacola, Florida); (5) ALmobile (Alabama); (6) LAmobile (east Louisiana and Mississippi); (7) LATXwest (west Louisiana and east Texas; (8) TXlaggal (central Texas); and (9) TXlagmat (south Texas). The descriptive region names in parentheses following each VDatum region names are used hereafter.

### Data analyses and presentation

We used the year of installation to determine the number of SET-MH stations installed within each state by year. For data presentation purposes, we determined the number of SET-MH stations within categorical bins defined for each parameter of interest. We present vertical position in 0.25-m elevation bins. Relative sea-level rise rate data are presented within the following five categories used in Thieler and Hammer-Klose (55): greater than 3.4, 3.0 to 3.4, 2.5 to 3.0, 1.8 to 2.5, and less than 1.8 mm/yr. Minimum air temperature and mean annual precipitation data were categorized into 3°C and 200 mm bins, respectively. Maps and geospatial datasets were created using Esri ArcGIS 10.3.1 (Environmental Systems Research Institute, Inc., Redlands, CA, USA). All other figures were created using Sigma Plot Version 12.0 (Systat Software, Inc., San Jose, CA, USA).

## Results

**Fig 2:**
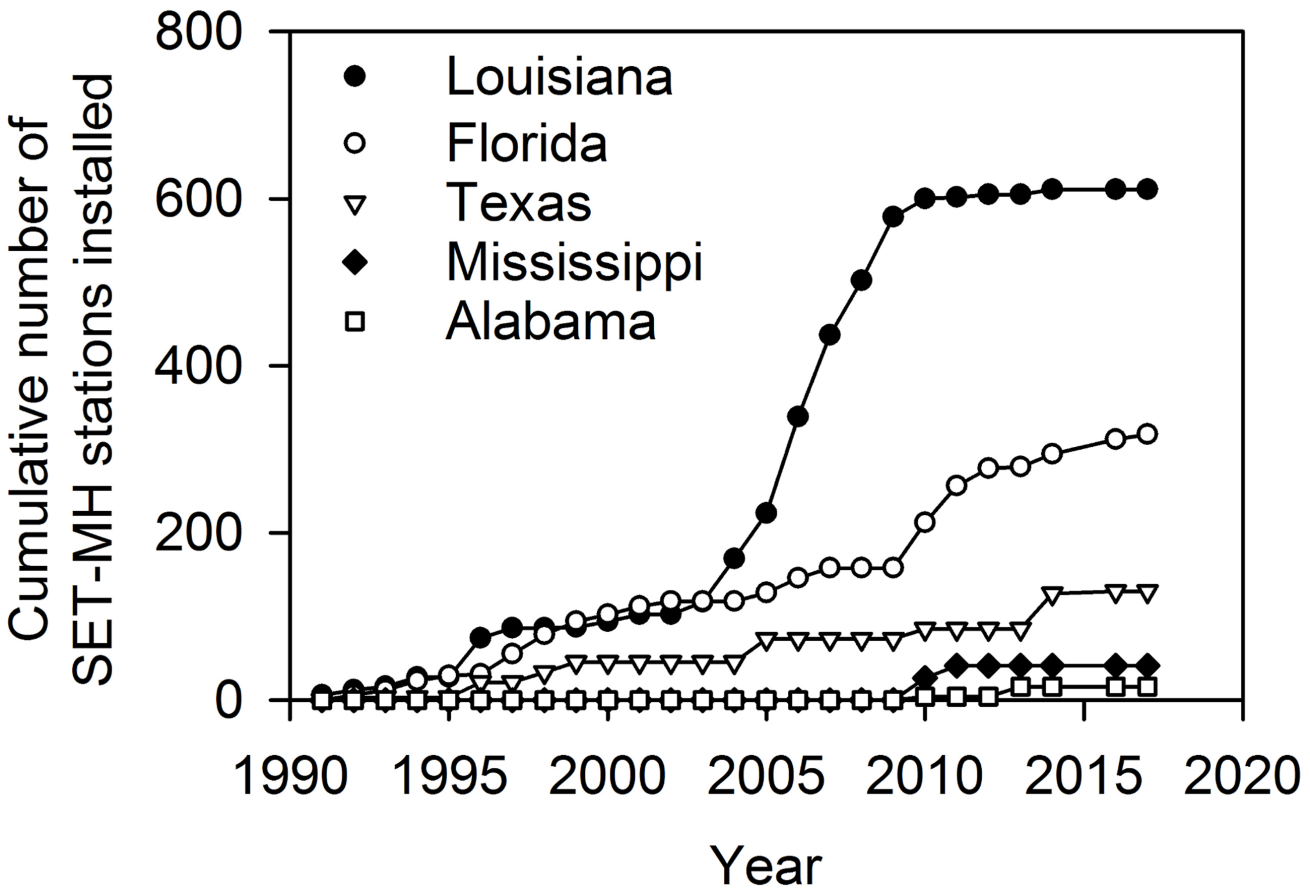
The temporal distribution of SET-MH station installations in U.S. Gulf of Mexico states.

We identified 1116 SET-MH stations in the region managed by various federal, state, and university-affiliated scientists. The State of Louisiana had the greatest number of stations (611, 55% of the total) followed by Florida (318, 28% of the total), Texas (130, 12% of the total), Mississippi (41, 4% of the total), and Alabama (16, 1% of the total) (Figs 1 and 2). The SET-MH approach was developed in Louisiana in the 1990s [24, 31], and some SET-MH stations in Louisiana, Florida, and Texas were installed in the 1990s. However, the majority of stations in Louisiana were installed between 2006 and 2010 (Fig 2). Stations in Mississippi and Alabama have been installed since 2010.

**Fig 3:**
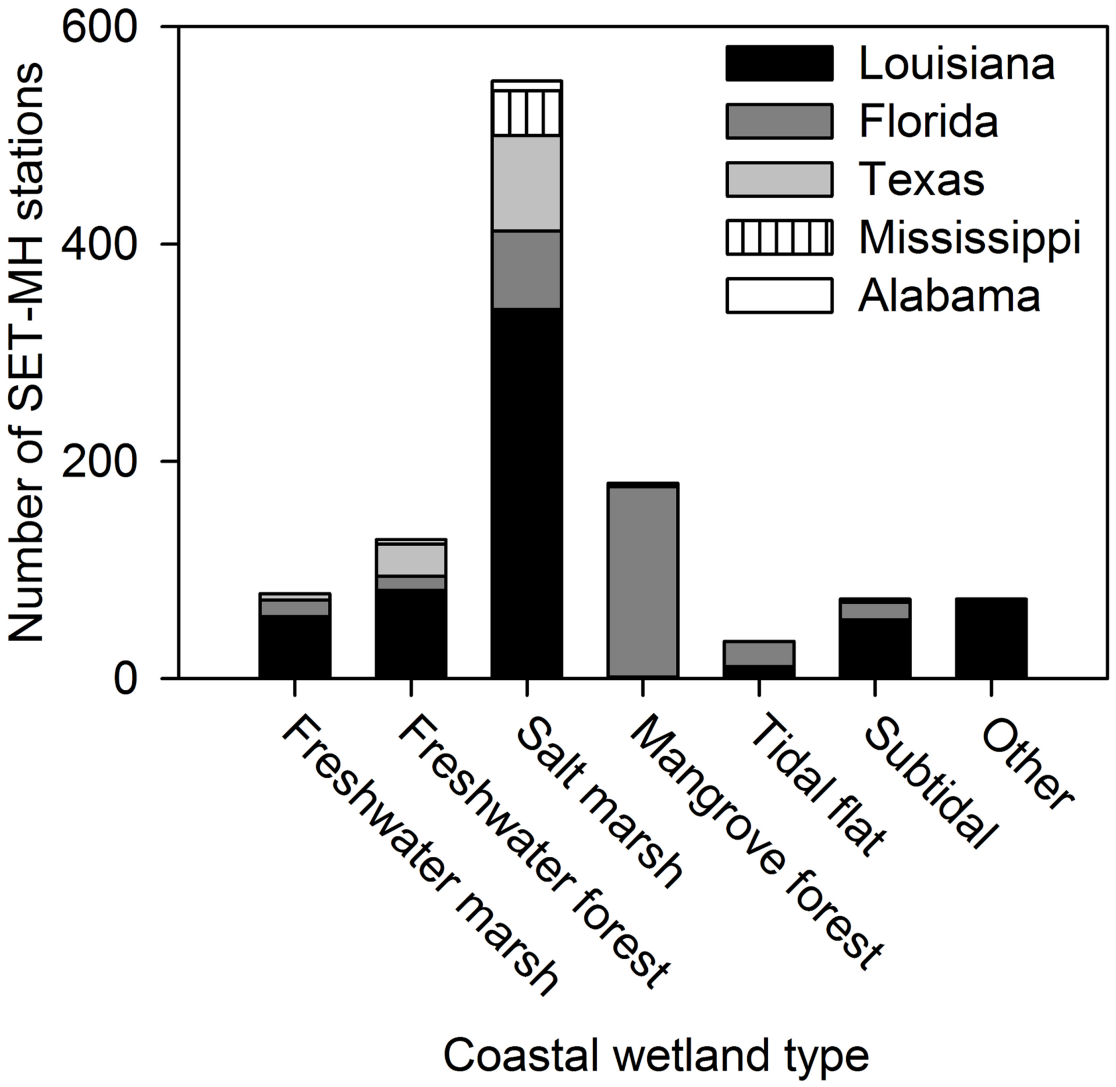
The distribution of SET-MH stations within coastal wetland types and states.

The majority of the SET-MH stations in the region are located in salt marshes (550, 49% of the total), followed by mangrove forests (180, 16% of the total), freshwater forested wetlands (128, 11% of the total), freshwater marshes (78, 7% of the total), subtidal habitats (73, 7% of the total), and tidal flats (34, 3% of the total) (Figs 1A and 3). Other types of habitat (i.e., riverine and lacustrine wetlands and other unclassified habitats) contain 73 stations (7% of the total).

**Fig 4:**
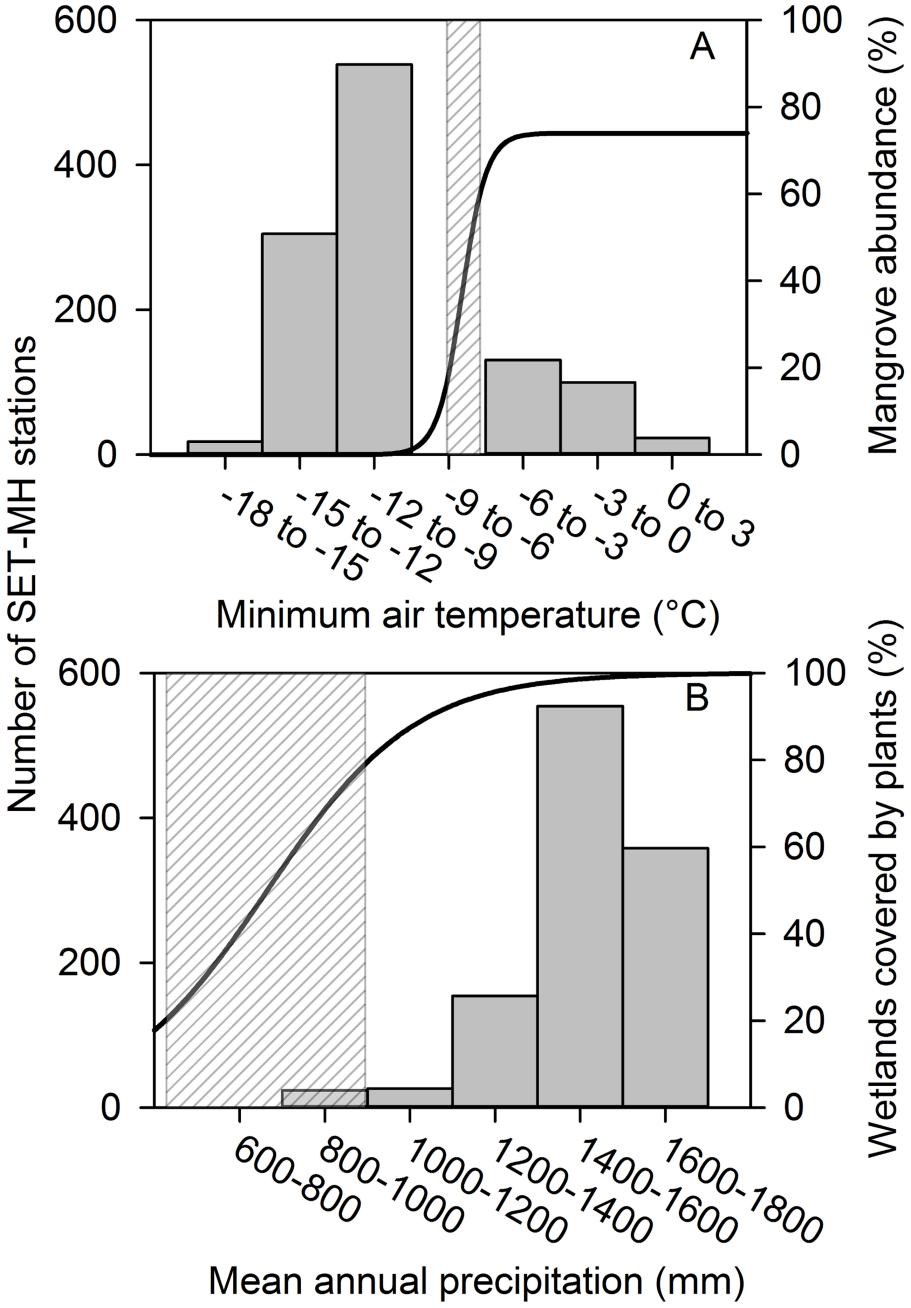
The distribution of SET-MH stations across ecologically-relevant climatic gradients. (A) minimum air temperature and (B) mean annual precipitation. SET-MH stations are shown via the vertical solid gray bars and left y axis. For interpretation within the context of coastal wetland transition zones, we also present established sigmoidal relationships between: (1) minimum temperature and the abundance of mangrove forests (solid black line and right y axis in A) [44]; and (2) mean annual precipitation and the coverage of wetland plants (solid black line and right y axis in B) [45]. Temperature and precipitation threshold zones are illustrated by the rectangles with light gray diagonal hatch lines in A and B, respectively.

With regards to air temperature regimes, the majority of the SET-MH stations (862, 78% of the total) are installed in wetlands that are cold enough to support salt marshes and freeze-tolerant vegetation; fewer stations (245, 23% of the total) are installed in areas with winter air temperature regimes that are warm enough to support freeze-sensitive mangrove forests (Figs 1B and 4A). With respect to precipitation regimes, the majority of the SET-MH stations (1092, 98% of the total) are installed in areas that are wet enough to support lower salinities and a high coverage of wetland plants; fewer stations (24, 2% of the total) are installed in low-rainfall areas that are dry and salty enough to support a low coverage of wetland plants (i.e., areas where hypersaline conditions lead to expansive salt flats) (Figs 1C and 4B). To aid with the interpretation of SET-MH distribution relative to climate, we present established sigmoidal relationships between: (1) minimum temperature and the abundance of mangrove forests [44] (line in Fig 4A); and (2) mean annual precipitation and the coverage of wetland plants [45] (line in Fig 4B).

**Fig 5:**
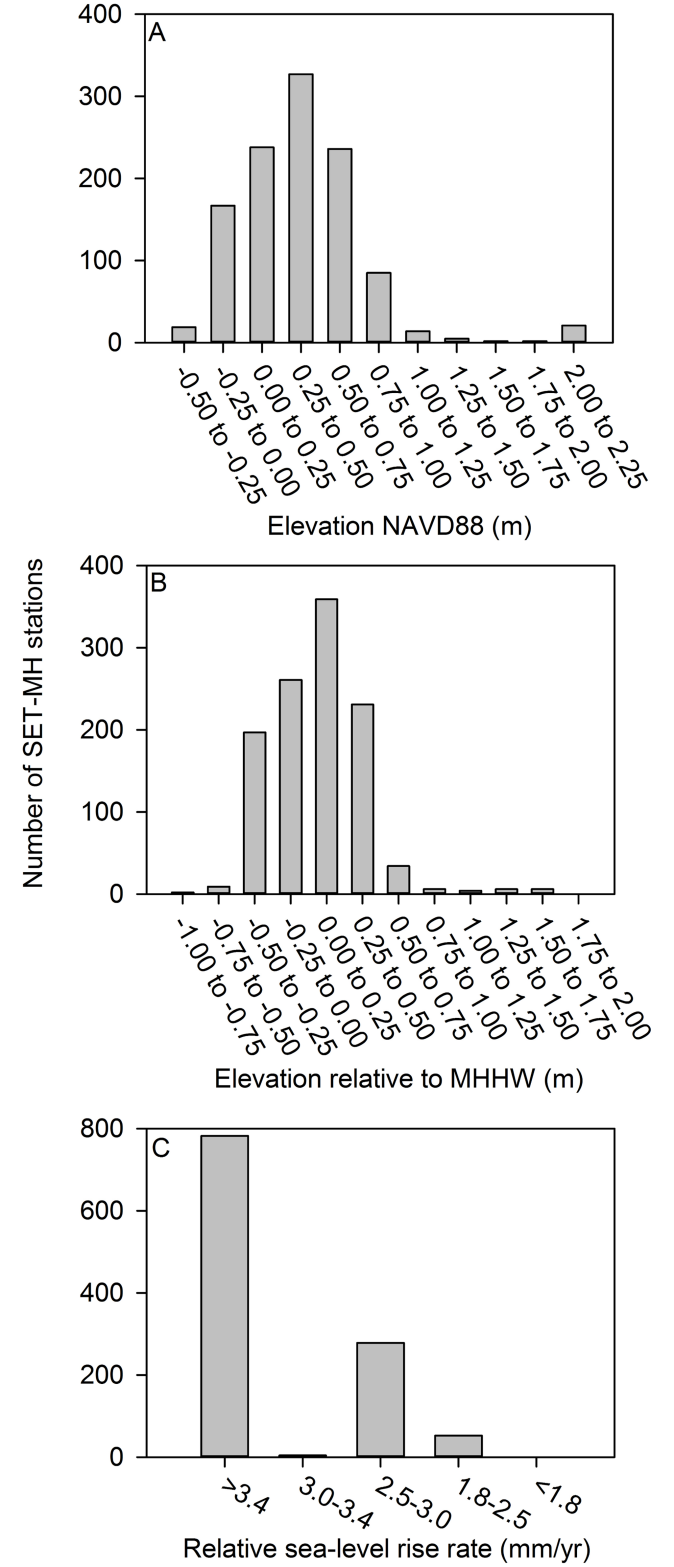
The distribution of SET-MH stations across elevation and inundation gradients. (A) elevation, (B) elevation relative to mean higher high water (MHHW), and (C) rate of relative sea-level rise.

Lidar-derived elevation category assignments show that the majority of the SET-MH stations (1053, 94% of the total) are located between -0.25 and 1.00 m NAVD88, with the highest number (327, 29% of the total) located between 0.25 and 0.50 m NAVD88 (Figs 1D and 5A). Tidal datum-derived elevation data indicate that a majority of stations (1048, 94% of the total) are located between -0.50 and 0.50 m elevation relative to MHHW, with the highest number (359, 32% of the total) installed between 0.00 and 0.25 m elevation relative to MHHW (Figs 1E and 5B). According to the relative sea-level rise grouping data contained within the USGS Coastal Vulnerability Index, the majority of SET-MH stations (782; 70% of the total) are installed in areas with relative sea-level rise rates greater than 3.4 mm/yr (Figs 1F and 5C).

**Fig 6:**
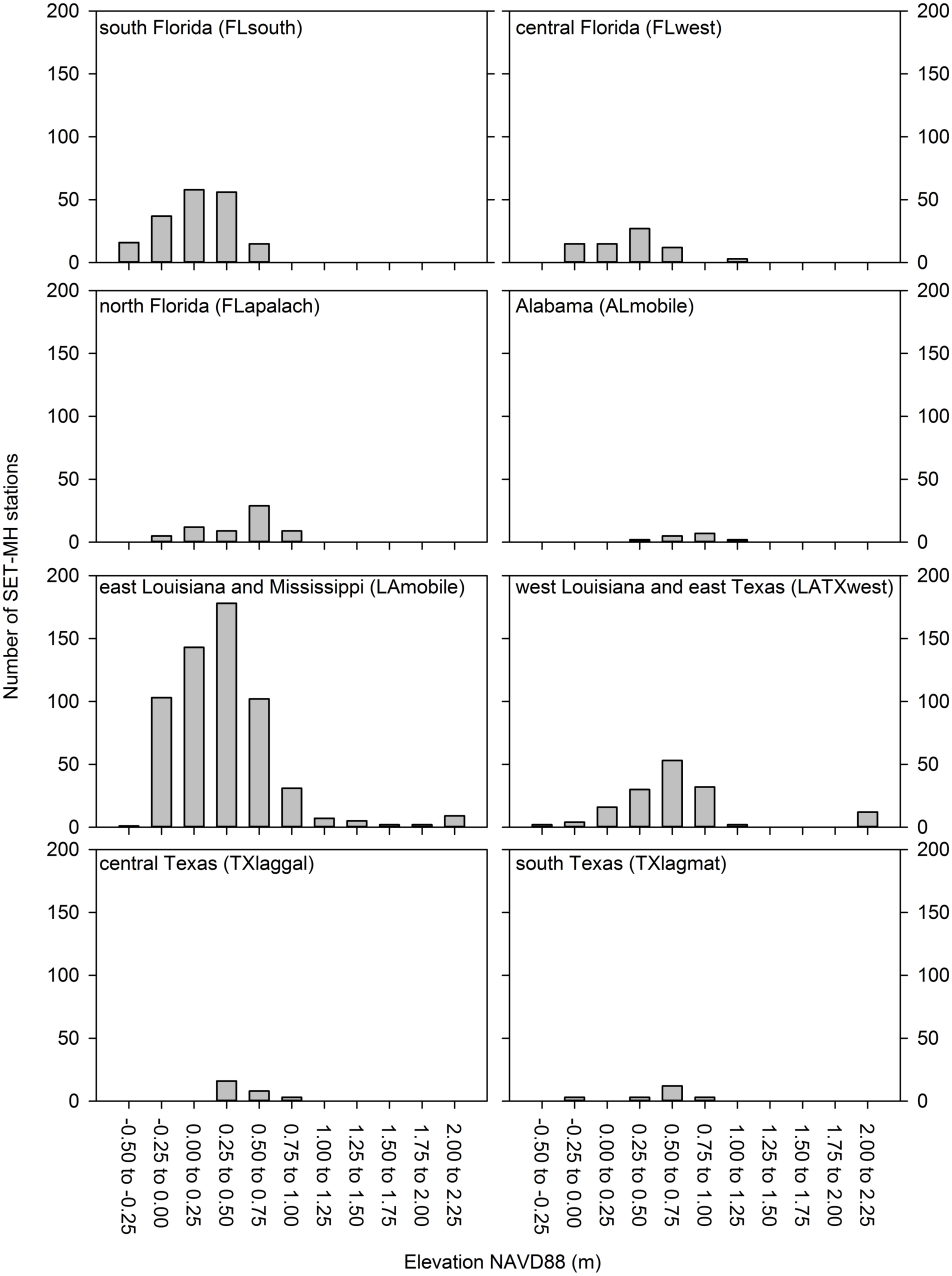
The distribution of SET-MH stations across elevation gradients within eight tidal datum regions. The Pensacola, Florida tidal datum region is not shown because it does not have any SET-MH stations.

Evaluation of station distribution within tidal datum regions revealed that the east Louisiana and Mississippi VDatum region has the highest number of SET-MH stations (583, 52% of the total; Fig 6). The south Florida and the west Louisiana/east Texas VDatum regions have the second and third highest number of stations (182 and 151 stations, 16 and 14% of the total, respectively). Station distribution in these three VDatum regions resembles a bell-shaped curve with a peak at the intertidal elevations where tidal saline wetlands are most abundant (Fig 6). The central Florida and north Florida VDatum regions have 72 and 64 stations, respectively (6 and 6% of the total, respectively). The number of SET-MH stations in the remaining VDatum regions are low (Fig 6), with one of the nine VDatum regions (Pensacola, Florida) containing no stations.

## Discussion

Climate change, accelerated sea-level rise, and growing human populations are expected to transform coastal landscapes across the globe in the coming century. As the stability and fate of coastal wetlands becomes more uncertain, there is a pressing need for information regarding coastal wetland surface elevation change relative to sea level. Such information is needed to assess vulnerability and inform management decisions. Here, we present the results of a regional inventory of SET-MH stations for the northern Gulf of Mexico (USA). We characterize the spatial and temporal distribution of SET-MH stations with an emphasis on the distribution across ecologically-relevant abiotic gradients. While our analyses highlight areas with high densities of SET-MH stations, they also reveal critical data gaps.

### Distribution of SET-MH stations across abiotic gradients and wetland types

Coastal wetland surface elevation change and responses to sea-level rise are greatly influenced by abiotic factors (e.g., inundation, salinity), which affect plant survival and growth [10, 16, 17, 56]. For example, Morris et al. [10] showed that salt marsh plant productivity varies across elevation gradients and that coastal wetland stability in the face of sea-level rise is dependent upon feedbacks between inundation, plant performance, and sedimentation (see also: [16, 57, 58–60]). The elevation-productivity relationship identified by Morris et al. [10] stemmed from productivity measurements that spanned an inundation gradient (see also: [61]), and these measurements contributed to the development of a seminal model of coastal wetland response to sea-level rise. Although many other examples exist, we highlight this single example to illustrate the tremendous value of strategic measurements made across ecologically-relevant abiotic gradients.

From a modeling and monitoring perspective, there is a need for temporally relevant surface elevation change data that span important abiotic gradients [10, 16, 57, 58]. Biogeomorphic feedbacks and nonlinear relationships are common in coastal wetlands; hence, our understanding of coastal wetland responses to sea-level rise can be improved by models that are developed from data that fully span critical abiotic gradients and relevant time scales. By original design, SET-MH stations have historically been installed by scientists seeking data to address specific research questions, often via comparison of categorical treatments (e.g., wetland type differences) at local scales. As a result, SET-MH stations typically are not systematically distributed across abiotic gradients, and temporal records can be disjunct. Louisiana is an exceptional case with numerous SET-MH stations spanning broad elevation, inundation, and salinity gradients [21, 23, 62]. For most coastal regions in the northern Gulf of Mexico (i.e., all other VDatum regions in Fig 6), SET-MH stations do not fully or adequately span elevation gradients. From a modeling and monitoring perspective, there is a need in many coastal areas for long-term records from additional SET-MH stations that are systematically and strategically installed across elevation, salinity, inundation, tidal range, sediment supply, subsidence, relative sea-level rise, wetland type, and landscape position gradients. The resultant data would allow scientists to better calibrate and validate models of future coastal wetland change, including their response to sea-level rise. In addition to a need for data that span estuary-scale gradients (e.g., salinity, landscape position), there is also much potential to take advantage of local gradients within a single wetland. For example, SET-MH measurements collected across ecologically-important elevation gradients within a single wetland could be used to better characterize local linkages between elevation, inundation regime, sedimentation, and net surface elevation change.

In addition to gradients in elevation and inundation, one of our goals was to assess the distribution of SET-MH stations across coastal wetland types and ecologically-relevant air temperature gradients. Along the Gulf of Mexico coast, the distribution of mangrove forests relative to salt marshes is largely controlled by winter air temperature regimes. Freezing temperatures can lead to mangrove damage or mortality [63–66]; hence, mangrove forests are restricted to tropical and subtropical climates [47, 67]. In contrast, salt marsh graminoids (i.e., grasses, sedges, and rushes) are freeze-tolerant and dominate coastal wetlands located in the colder and more northern coastal reaches of the Gulf of Mexico [2, 44, 46]. Here, we evaluated the distribution of SET-MH stations across the air temperature gradient that governs the relative dominance of mangrove forests versus salt marshes. In the southeastern USA, there is a positive sigmoidal relationship between minimum air temperatures and mangrove dominance relative to salt marshes [44]. Our analyses show that whereas there are a large number of SET-MH stations installed in the colder salt marsh-dominated coastal reaches, there are fewer stations located in the warmer mangrove-dominated coastal areas. Even more importantly, very few SET-MH stations are installed within or near the temperature-based threshold zone that separates mangrove forests from salt marshes (i.e., near the temperature threshold in Fig 4A; -7.6 to -6.3°C). In this threshold zone, small changes in temperature can lead to abrupt changes in wetland ecosystem structure and function (i.e., mangrove forest expansion at the expense of salt marsh) [44, 46]. This zone is expected to shift northward due to climate change [44, 66]. Given the potential for climate change-induced mangrove expansion in parts of Florida, Louisiana, and Texas [2, 44, 68], there is a pressing need to better understand the implications of mangrove encroachment for wetland elevation change and the ability of coastal wetlands to keep pace with rising sea levels. Additional long-term data from SET-MH stations within the mangrove-to-marsh transition zone would provide scientists with critical information needed to better advance understanding of this issue.

We also assessed the distribution of SET-MH stations across ecologically-relevant precipitation gradients. In coastal wetlands, low precipitation and high evaporation can concentrate oceanic salts and produce hypersaline conditions [69], which are inhospitable to many coastal wetland plants. Along the Gulf of Mexico and elsewhere, precipitation and other freshwater inputs greatly influence the coverage of coastal wetland plants [47, 70–73]. Low rainfall, hypersaline conditions, and large expanses of unvegetated tidal saline wetlands are common in south Texas, particularly within the Laguna Madre Estuary [2, 69]. Within the northwestern portion of the Gulf of Mexico, there is a sigmoidal relationship between mean annual precipitation and the coverage of foundation plant species in tidal saline wetlands [45]. As part of this inventory, we sought to characterize the distribution of SET-MH stations across that precipitation-plant coverage gradient. We found that very few SET-MH stations are installed in the drier sections of the Gulf of Mexico; most stations are installed in coastal zones that have sufficient rainfall to prompt dominance of salt marshes or mangrove forests (Fig 4B). Near the precipitation-based threshold zone, small changes in precipitation, freshwater availability, and/or sea level are expected to alter salinity regimes and lead to abrupt and large changes in the abundance of coastal wetland plants [2, 45–47, 72, 74–78]. Coastal wetland plant performance greatly influences surface elevation change and the ability of coastal wetlands to keep pace with sea-level rise. Within this context, there is a pressing need to better understand the implications of salinity-induced changes in plant performance on the capacity of coastal wetlands to adjust to rising sea levels. Our inventory highlights the need for SET-MH stations and data in the drier portions of the study area (i.e., south and central Texas). Our wetland-type analyses also support this conclusion; there are very few SET-MH stations installed in salt flats compared to salt marshes and mangrove forests.

### Gulf of Mexico wetlands are diverse

Most models of coastal wetland response to sea-level rise in the USA have historically focused on marshes dominated by *Spartina alterniflora* [10, 56, 57]. However, the diversity of plant communities in coastal wetlands along the Gulf of Mexico coast is high, and *S*. *alterniflora* is just one of many common wetland foundation plant species. Tidal salt marshes in the region can be dominated by succulent plant species [79] or other graminoid species (e.g., *Juncus roemerianus* or *S*. *patens*) [59, 80]. Moreover, three different mangrove species are common in the region [81], and unvegetated salt flats covered by algal mats are abundant in areas with low rainfall and hypersaline conditions [2, 69]. Given the diversity of wetland plant communities in the region, there is a need to explicitly consider the impact of these different plant species and functional groups upon surface elevation change and coastal wetland response to rising sea levels. Differences in landscape setting also merit additional attention [82]; in recent reviews, Krauss et al. [14] and Woodroffe et al. [15] highlight the importance of considering differences in biogeomorphic processes in different landscape settings. Hydrologic regimes, sediment supply, and subsidence rates vary greatly in the region and warrant more attention [23]. In our analyses, we focused only on a handful of categories (e.g., wetland types) and abiotic gradients (e.g., elevation, temperature, precipitation, relative sea-level rise) that could be easily assessed using geospatial data. However, coastal wetland biogeomorphic and plant community zonation patterns across the Gulf of Mexico are diverse [2, 46, 81–84] and not adequately covered by currently available geospatial data. There are many more categories and gradients that are important to include within the context of elevation change in coastal wetlands. The strategic development of a regional SET-MH network could help incorporate and address the importance of such gradients.

### Wetland loss and SET-MH stations in Louisiana

Coastal wetland loss has been very high in the Mississippi River Deltaic Plain (MRDP) of coastal Louisiana due to a combination of natural and anthropogenic factors [85–87]. The rate of relative sea-level rise in parts of the MRDP can exceed 2 cm/year, due in part to high rates of subsidence and low rates of accretion [23]. Since 1932, Louisiana has lost more than ~4,900 km^2^ of land [88]. In many ways, the rapid pace of coastal wetland loss in Louisiana is what prompted the development, refinement, and widespread adoption of the SET [24, 26, 40] and, subsequently, the SET-MH approach [25, 27, 31]. The urgency to measure wetland surface elevation change using the SET-MH methodology is much greater in Louisiana compared to other coastal regions because of high rates of relative sea-level rise, high rates of wetland loss via submergence and conversion to open water, and high variability in SET-MH measurements [21, 23]. The high rate of wetland submergence underscores the need to initiate and sustain measurements now to develop longer-term records before more wetlands are lost. Our relative sea-level rise analyses support this statement; as expected, the number of SET-MH stations is highest in coastal regions with high rates of relative sea-level rise (i.e., Louisiana; Fig 5C). In this regard and given the tremendous amount of data available, Louisiana serves as a natural laboratory for investigating the effects of accelerated sea-level rise on coastal wetlands [23, 89].

SET and SET-MH-based research in Louisiana began with individual scientists who needed high-resolution surface elevation change measurements to address specific research questions related to coastal wetland degradation and loss. However, the number of SET-MH stations greatly increased with the creation of the Coastwide Reference Monitoring System (CRMS) [62]. CRMS was designed as a network of sites, within the Louisiana coastal zone, for monitoring the performance of coastal wetland restoration efforts implemented under the Coastal Wetlands Planning, Protection, and Restoration Act (CWPPRA) of 1990. Funding for CRMS was authorized by the CWPPRA Task Force in 2003, which led to the installation of most of the CRMS affiliated SET-MH stations in 2006. Of the 611 SET-MH stations in Louisiana, 332 (54%) are associated with CRMS monitoring stations. Due primarily to CRMS, we suspect that Louisiana has the highest SET-MH station density over a broad geographical scale of any SET-MH network in the world [20, 21]. As a result, the distribution of SET-MH stations across wetland types and ecologically-relevant abiotic gradients is much higher in Louisiana than in other parts of the northern Gulf of Mexico. Much of the SET-MH data from CRMS is now 10 years old and these SET-MH data are used to assess and model coastal wetland vulnerability. For example, Stagg et al. [21] developed a Submergence Vulnerability Index for CRMS sites, which uses hydrologic and SET-MH data to determine the vulnerability of Louisiana’s coastal wetlands based on their ability to adjust to sea-level rise. Jankowski et al. [23] used CRMS SET-MH data from 274 sites to compare vertical accretion rates with relative sea-level rise rates within wetlands in Louisiana’s Mississippi Delta and Chenier Plain; their analyses show that many of Louisiana’s wetlands in Louisiana are not keeping pace with current rates of relative sea-level rise (i.e., 35 and 42% of wetlands in the Mississippi Delta and Chenier Plain, respectively, are not keeping pace). The CRMS data have also been incorporated into models of wetland biogeomorphic responses to various restoration alternatives under Louisiana’s Comprehensive Master Plan for a Sustainable Coast [90]. The Louisiana CRMS network serves as a valuable example of how a coordinated and extensive SET-MH network can inform wetland science, restoration, and management needs.

### SET-MH stations in Florida, Texas, Alabama, and Mississippi

The number of SET-MH stations in south Florida is also relatively high due to research efforts aimed at quantifying elevation change in wetlands within and near the Everglades [12, 30, 33, 91–93]. Some of the south Florida SET-MH stations were installed in the 1990s. As a result, multiple SET types are still in use [i.e., the original SET, deep RSET, and shallow RSET], and some sites have an impressive ~20-year record of SET-MH data [30, 91, 94]. Other SET-MH stations in Florida are concentrated near the Faka Union Canal, Blackwater River, Rookery Bay National Estuarine Research Reserve [12, 92], Ten Thousand Islands National Wildlife Refuge [33], J.N. Ding Darling National Wildlife Refuge, Florida Keys, Tampa Bay, St. Marks National Wildlife Refuge, and the Apalachicola National Estuarine Research Reserve.

In Texas, most of the SET-MH stations are installed along the northern and central coasts. Stations are concentrated within several National Wildlife Refuges and the Mission-Aransas National Estuarine Research Reserve. To our knowledge, there are no SET-MH stations installed in south Texas in the Laguna Madre estuary. In Mississippi and Alabama, the Grand Bay and Weeks Bay National Estuarine Research Reserves have developed long-term SET-MH monitoring stations. The number of SET-MH stations in Alabama and Mississippi are relatively low, and many stations were installed within approximately the last five years. However, please note that the coastlines of Alabama and Mississippi are comparatively smaller than the coastlines of Texas, Louisiana, and Florida.

### Beyond just the SET-MH method: Limitations and integration with other approaches

In this communication, our intent is not to imply that the SET-MH method is the only method for assessing coastal wetland vulnerability to sea-level rise. There are many different approaches for assessing coastal wetland vulnerability to sea-level rise, and each approach has strengths and weaknesses. Multiple approaches could be integrated into a regional network for the Gulf of Mexico. For example, marsh organs [95, 96] could be used to quantify relationships between inundation and productivity of different plant species (e.g., succulents, graminoids, mangroves, and microbial mats) [97–100], which can be incorporated into models of wetland response to sea-level rise [59, 101]. Similarly, larger-scale *in situ* manipulations via weirs could be used to elucidate biogeomorphic responses to inundation [102]. There are also opportunities to develop novel experimental designs at restoration sites to improve our understanding of ecological responses to inundation, sedimentation, and saltwater intrusion [33, 35, 103, 104].

Despite the many benefits of the SET-MH approach, the method does have several limitations that should be considered. In addition to the spatial data gaps identified in this inventory, temporal data gaps exist for SET-MH stations if the local historical and geological contexts are unknown. Radiometrically-dated soil cores can help bridge the gap, and have been used to quantify accretion rates over decadal to millennial timescales in marsh and mangrove wetlands in the northern Gulf of Mexico (e.g., [105, 106–110]). However, rates of elevation change and radiometric accretion are often different (e.g., [92]), as are accretion rates measured using different radiometric methods or timescales [111–113]. Interpreting the reasons for these differences can be enhanced by using analytical tracers (e.g., organic matter or mineral content, sediment grain size, stable isotopes, elemental ratios, and other sedimentary biomarkers) to provide insights into the biogeochemical processes that contribute to soil formation, loss, and surface elevation change. A regional-scale analysis of the distribution of radiometrically-dated soil core records could inform questions related to where the greatest differences or similarities exist between methods and timescales in different wetland habitats or across abiotic gradients. Soil core bulk density and organic matter data can also be incorporated into vertical accretion models. For example, Morris et al. [114] recently used data from 5075 soil samples collected from coastal wetlands across the contiguous United States to develop a mixing model that can be used to evaluate maximum steady state vertical accretion rates as well as the potential contributions of organic and inorganic matter.

The requirement for long-term data (e.g., at least 5–10 years) is another potential limitation of the SET-MH approach. Many stations in the network were established with short-term funds to address a specific research question, not to serve as a long-term monitoring platform. The strategic collection of short-term, high temporal resolution SET-MH data can be valuable and help elucidate the effects of seasonal processes and extreme events (e.g., hydrologic fluctuations and hurricanes) [30, 91]. However, the collection of long-term data records requires considerable time, resources, and organizational stability. Unfortunately, data collection at some SET-MH stations was discontinued when initial project funds ran out, or scientists retired, transitioned to new positions, or adjusted research priorities. Some of the SET-MH stations included in our analyses are currently inactive for these reasons. Although certain stagnant SET-MH stations could be reactivated, some of the older SET-MH stations may no longer be functional or available for incorporation into a regional network. Hence, the development and maintenance of a regional SET-MH network would require additional resources and organizational capacity to maintain and coordinate the currently-available SET-MH stations as well as incorporate new SET-MH stations. While additional sites must be added in certain areas, the first priority of a regional network should be to evaluate existing sites and make sure that they are well maintained and continuously recorded.

Another limitation of the SET-MH approach is that the high vertical resolution data produced may only be representative of a relatively small spatial area. There are several complementary approaches that can be integrated with SET-MH data to expand the area of inference. For example, technological advancements [e.g., Real-Time Kinematic Global Navigation Satellite Systems (RTK-GNSS), Continuously Operating Reference Station (CORS) networks, airborne and terrestrial lidar] continue to improve and diversify options for measuring wetland elevations. Although other approaches do not match the high vertical resolution provided by SET-MH measurements [20], there are many reasons, including increased spatial coverage, for incorporating these complementary approaches into SET-MH network protocols and products. To make full use of the mm-level accuracy provided by SET-MH elevation change measurements, site-specific hydrologic contexts and rates of relative sea-level rise should be carefully considered and quantified to the same level of accuracy [23, 27]. See Cahoon [27] for a valuable discussion of the importance of co-locating tide gage and SET-MH stations and calculating wetland-specific relative sea-level rise rates using SET-derived elevation trends and tide gage-derived relative sea-level rise.

The general objectives for developing a regional SET-MH monitoring network could include the multi-scale assessment of wetland submergence potential under sea-level rise as well as the improved characterization of biogeomorphic processes that affect wetland elevations. Specific monitoring objectives should be identified by local and regional coastal resource managers and planners in coordination with coastal scientists to address specific environmental concerns (e.g., the CRMS network in Louisiana). Establishing a regional monitoring network capable of meeting these objectives will require: (1) careful screening of the existing SET-MH stations to ensure that appropriate comparisons can be made among stations; (2) strategic addition of SET-MH stations to complement the existing network where gaps are noted; (3) careful coordination of sampling protocols to optimize the long-term and robust collection of data that can be used to meet the stated goals of the network; and (4) the coordination and integration of SET-MH data collection efforts with other approaches for investigating coastal wetland vulnerability to sea-level rise.

In terms of organizational capacity and precedent, there are various potential models for how a regional monitoring network could be supported and maintained. While the potential funding mechanism and organizational structure for a regional network would need to be carefully considered, the following long-term monitoring programs could serve as examples to learn and build from: (1) CRMS in Louisiana; (2) USGS stream-gage network; (3) NOAA Water Level Observation Network (NWLON); (4) NSF Long-term Ecological Research (LTER) Program; and (5) National Ecological Observatory Network (NEON). A valuable next step would be to cultivate the resources and organizational capacity needed to plan, establish, and maintain a coordinated elevation change network for the Gulf of Mexico region.

### Conclusions

In the face of accelerated sea-level rise, there is an increasing need for elevation change networks that can contribute data required for regional ecological models and vulnerability assessments [13, 20, 23]. For modeling and monitoring purposes, there is a need for long-term data (e.g., greater than 10 years) from SET-MH stations that are strategically distributed across ecologically-relevant abiotic gradients at both local and regional scales. Collectively, our analyses provide the basis for the development of a coordinated and strategic regional elevation change network from the current unplanned collection of SET-MH stations. The regional network would provide data for predicting and preparing for the responses of coastal wetlands in the Gulf of Mexico region to accelerated sea-level rise and other aspects of global change.

## References

[1] McKee K, Rogers K, Saintilan N. Response of salt marsh and mangrove wetlands to changes in atmospheric CO_2_, climate, and sea level In: Middleton BA, editor. Global Change and the Function and Distribution of Wetlands: Global Change Ecology and Wetlands. Dordrecht, Netherlands: Springer; 2012 p. 63–96.

[2] Gabler CA, Osland MJ, Grace JB, Stagg CL, Day RH, Hartley SB, et al Macroclimatic change expected to transform coastal wetland ecosystems this century. Nature Climate Change. 2017;7:142–147.

[3] Enwright NM, Griffith KT, Osland MJ. Barriers to and opportunities for landward migration of coastal wetlands with sea-level rise. Frontiers in Ecology and the Environment. 2016;14(6):307–316.

[4] Doyle TW, Krauss KW, Conner WH, From AS. Predicting the retreat and migration of tidal forests along the northern Gulf of Mexico under sea-level rise. Forest Ecology and Management. 2010;259(4):770–777.

[5] Kirwan ML, Megonigal JP. Tidal wetland stability in the face of human impacts and sea-level rise. Nature. 2013;504(7478):53–60. doi: 10.1038/nature12856 24305148 10.1038/nature12856

[6] Barbier EB, Hacker SD, Kennedy C, Koch EW, Stier AC, Silliman BR. The value of estuarine and coastal ecosystem services. Ecological Monographs. 2011;81:169–193.

[7] Engle VD. Estimating the provision of ecosystem services by Gulf of Mexico coastal wetlands. Wetlands. 2011;31:179–193.

[8] Millennium Ecosystem Assessment. Ecosystems and human well-being: Synthesis. Washington, D.C.: Island Press; 2005.

[9] Richardson CJ. Ecological functions and human-values in wetlands—a framework for assessing forestry impacts. Wetlands. 1994;14(1):1–9.

[10] Morris JT, Sundareshwar PV, Nietch CT, Kjerfve B, Cahoon DR. Responses of coastal wetlands to rising sea level. Ecology. 2002;83(10):2869–2877.

[11] Cahoon DR, Hensel PF, Spencer T, Reed DJ, McKee KL, Saintilan N. Coastal wetland vulnerability to relative sea-level rise: wetland elevation trends and process controls In: Verhoeven JTA, Beltman B, Bobbink R, Whigham DF, editors. Wetlands and natural resource management. Berlin, Germany: Springer; 2006 p. 271–292.

[12] McKee KL. Biophysical controls on accretion and elevation change in Caribbean mangrove ecosystems. Estuarine, Coastal and Shelf Science. 2011;91:475–483.

[13] Lovelock CE, Cahoon DR, Friess DA, Guntenspergen GR, Krauss KW, Reef R, et al The vulnerability of Indo-Pacific mangrove forests to sea-level rise. Nature. 2015;526:559–563. doi: 10.1038/nature15538 26466567 10.1038/nature15538

[14] Krauss KW, McKee KL, Lovelock CE, Cahoon DR, Saintilan N, Reef R, et al How mangrove forests adjust to rising sea level. New Phytologist. 2014;202:19–34. doi: 10.1111/nph.12605 24251960 10.1111/nph.12605

[15] Woodroffe CD, Rogers K, McKee KL, Lovelock CE, Mendelssohn IA, Saintilan N. Mangrove sedimentation and response to relative sea-level rise. Annual Review of Marine Science. 2016;8:243–266. doi: 10.1146/annurev-marine-122414-034025 26407146 10.1146/annurev-marine-122414-034025

[16] Kirwan ML, Temmerman S, Skeehan EE, Guntenspergen GR, Fagherazzi S. Overestimation of marsh vulnerability to sea level rise. Nature Climate Change. 2016;6:253–260.

[17] Mendelssohn IA, Morris JT. Eco-physiological controls on the productivity of *Spartina alterniflora* Loisel In: Weinstein MP, Kreeger DA, editors. Concepts and Controversies in Tidal Marsh Ecology. Dordrecht, Netherlands: Kluwer Academic Publishers; 2000 p. 59–80.

[18] Perillo GME, Wolanski E, Cahoon DR, Brinson MM. Coastal wetlands: an integrated ecosystem approach. Amsterdam, Netherlands: Elsevier; 2009.

[19] Krauss KW, Lovelock CE, McKee KL, López-Hoffman L, Ewe SML, Sousa WP. Environmental drivers in mangrove establishment and early development: a review. Aquatic Botany. 2008;89(2):105–127.

[20] Webb EL, Friess DA, Krauss KW, Cahoon DR, Guntenspergen GR, Phelps J. A global standard for monitoring coastal wetland vulnerability to accelerated sea-level rise. Nature Climate Change. 2013;3:458–465.

[21] Stagg CL, Sharp LA, McGinnis TE, Snedden GA. Submergence Vulnerability Index development and application to Coastwide Reference Monitoring System Sites and Coastal Wetlands Planning, Protection and Restoration Act projects. U.S. Geological Survey, Open-File Report 2013–1163, 2013 2331–1258.

[22] Raposa KB, Wasson K, Smith E, Crooks JA, Delgado P, Fernald SH, et al Assessing tidal marsh resilience to sea-level rise at broad geographic scales with multi-metric indices. Biological Conservation. 2016;204:263–275.

[23] Jankowski KL, Törnqvist TE, Fernandes AM. Vulnerability of Louisiana’s coastal wetlands to present-day rates of relative sea-level rise. Nature Communications. 2017;8:14792 doi: 10.1038/ncomms14792 28290444 10.1038/ncomms14792 PMC5355890

[24] Cahoon DR, Lynch JC, Perez BC, Segura B, Holland RD, Stelly C, et al High-precision measurements of wetland sediment elevation: II. The rod surface elevation table. Journal of Sedimentary Research. 2002;72(5):734–739.

[25] Callaway JC, Cahoon DR, Lynch JC. The surface elevation table–marker horizon method for measuring wetland accretion and elevation dynamics In: DeLaune RD, Reddy KR, Richardson CJ, Megonigal JP, editors. Methods in Biogeochemistry of Wetlands. Madison, Wisconsin: Soil Science Society of America; 2013 p. 901–917.

[26] Cahoon DR, Lynch JC, Hensel P, Boumans RMJ, Perez BC, Segura B, et al High-precision measurements of wetland sediment elevation: I. Recent improvements to the sedimentation-erosion table. Journal of Sedimentary Research. 2002;72(5):730–733.

[27] Cahoon DR. Estimating relative sea-level rise and submergence potential at a coastal wetland. Estuaries and Coasts. 2015;38(3):1077–1084.

[28] Lynch JC, Hensel P, Cahoon DR. The surface elevation table and marker horizon technique: A protocol for monitoring wetland elevation dynamics. Natural Resources Report NPS/NCBN/NRR-2015/1078. Fort Collins, Colorado: National Park Service, 2015.

[29] Cahoon DR, Hensel P, Rybczyk J, McKee KL, Proffitt CE, Perez BC. Mass tree mortality leads to mangrove peat collapse at Bay Islands, Honduras after Hurricane Mitch. Journal of Ecology. 2003;91(6):1093–1105.

[30] Whelan KRT, Smith TJ III, Cahoon DR, Lynch JC, Anderson GH. Groundwater control of mangrove surface elevation: Shrink and swell varies with soil depth. Estuaries and Coasts. 2005;28(6):833–843.

[31] Cahoon DR, Reed DJ, Day JW. Estimating shallow subsidence in microtidal salt marshes of the southeastern United States: Kaye and Barghoorn revisited. Marine Geology. 1995;128:1–9.

[32] Rogers K, Saintilan N, Cahoon D. Surface elevation dynamics in a regenerating mangrove forest at Homebush Bay, Australia. Wetlands Ecology and Management. 2005;13(5):587–598.

[33] Howard RJ, Day RH, Krauss KW, From AS, Allain L, Cormier N. Hydrologic restoration in a dynamic subtropical mangrove-to-marsh ecotone. Restoration Ecology. 2017;25:471–482.

[34] Krauss KW, Cahoon DR, Allen JA, Ewel KC, Lynch JC, Cormier N. Surface elevation change and susceptibility of different mangrove zones to sea-level rise on Pacific high islands of Micronesia. Ecosystems. 2010;13(1):129–143.

[35] Baustian JJ, Mendelssohn IA, Hester MW. Vegetation's importance in regulating surface elevation in a coastal salt marsh facing elevated rates of sea level rise. Global Change Biology. 2012;18(11):3377–3382.

[36] Graham SA, Mendelssohn IA. Coastal wetland stability maintained through counterbalancing accretionary responses to chronic nutrient enrichment. Ecology. 2014;95(12):3271–3283.

[37] Lane RR, Day JW Jr, Day JN. Wetland surface elevation, vertical accretion, and subsidence at three Louisiana estuaries receiving diverted Mississippi River water. Wetlands. 2006;26(4):1130–1142.

[38] Schoot PM, de Jong JEA. Sedimentatie en erosie metingen met behulp van de Sedi-Eros-Tafel (Set) Notitie DDMI-82.401. Netherlands: Rijkswaterstaat; 1982.

[39] van Eerdt MM. The influence of vegetation on erosion and accretion in salt marshes of the Oosterschelde, The Netherlands. Vegetatio. 1985;62(62):367–373.

[40] Boumans RMJ, Day JW. High precision measurements of sediment elevation in shallow coastal areas using a sedimentation-erosion table. Estuaries and Coasts. 1993;16(2):375–380.

[41] Ward RD, Friess DA, Day RH, MacKenzie RA. Impacts of climate change on mangrove ecosystems: a region by region overview. Ecosystem Health and Sustainability. 2016;2:e01211.

[42] Cowardin LM, Carter V, Golet FC, LaRoe ET. Classification of wetlands and deepwater habitats of the United States, Report FWS/OBS-79/31. Washington, D.C.: U.S. Fish and Wildlife Service; 1979.

[43] Daly C, Halbleib M, Smith JI, Gibson WP, Doggett MK, Taylor GH, et al Physiographically sensitive mapping of climatological temperature and precipitation across the conterminous United States. International Journal of Climatology. 2008;28(15):2031–2064.

[44] Osland MJ, Enwright N, Day RH, Doyle TW. Winter climate change and coastal wetland foundation species: salt marshes vs. mangrove forests in the southeastern United States. Global Change Biology. 2013;19(5):1482–1494. doi: 10.1111/gcb.12126 23504931 10.1111/gcb.12126

[45] Osland MJ, Enwright N, Stagg CL. Freshwater availability and coastal wetland foundation species: ecological transitions along a rainfall gradient. Ecology. 2014;95(10):2789–2802. doi: 10.1890/13-1269.1

[46] Osland MJ, Enwright NM, Day RH, Gabler CA, Stagg CL, Grace JB. Beyond just sea-level rise: considering macroclimatic drivers within coastal wetland vulnerability assessments to climate change. Global Change Biology. 2016;22(1):1–11. doi: 10.1111/gcb.13084 26342186 10.1111/gcb.13084

[47] Osland MJ, Feher LC, Griffith KT, Cavanaugh KC, Enwright NM, Day RH, et al Climatic controls on the global distribution, abundance, and species richness of mangrove forests. Ecological Monographs. 2017;87:341–359.

[48] Medeiros S, Hagen S, Weishampel J, Angelo J. Adjusting lidar-derived digital terrain models in coastal marshes based on estimated aboveground biomass density. Remote Sensing. 2015;7(4):3507–3525.

[49] Su J, Bork E. Influence of vegetation, slope, and lidar sampling angle on DEM accuracy. Photogrammetric Engineering & Remote Sensing. 2006;72(11):1265–1274.

[50] Kidwell DM, Dietrich JC, Hagen SC, Medeiros SC. An Earth's Future Special Collection: Impacts of the coastal dynamics of sea level rise on low-gradient coastal landscapes. Earth's Future. 2017;5(1):2–9.

[51] Buffington KJ, Dugger BD, Thorne KM, Takekawa JY. Statistical correction of lidar-derived digital elevation models with multispectral airborne imagery in tidal marshes. Remote Sensing of Environment. 2016;186:616–625.

[52] Marcy D, Herold N, Waters K, Brooks W, Hadley B, Pendleton M, et al New mapping tool and techniques for visualizing sea level rise and coastal flooding impacts In: Wallendorf L, Jones C, Ewing L, Battalio B, editors. Solutions to coastal disasters. Anchorage, Alaska, USA: American Society of Civil Engineers; 2011 p. 474–490.

[53] Parker BB. The difficulties in measuring a consistently defined shoreline- the problem of vertical referencing. Journal of Coastal Research. 2003;38:44–56.

[54] Cooper HM, Fletcher CH, Chen Q, Barbee MM. Sea-level rise vulnerability mapping for adaptation decisions using LiDAR DEMs. Progress in Physical Geography. 2013;37(6):745–766.

[55] Thieler ER, Hammer-Klose ES. National assessment of coastal vulnerability to future sea level rise: preliminary results for the U.S. Gulf of Mexico coast: U.S. Geological Survey, Open-File Report 00–179, 1 sheet.; 2000.

[56] Kirwan ML, Murray AB. A coupled geomorphic and ecological model of tidal marsh evolution. Proceedings of the National Academy of Sciences. 2007;104(15):6118–6122. 10.1073/pnas.0700958104 PMC1851060 17389384

[57] Fagherazzi S, Kirwan ML, Mudd SM, Guntenspergen GR, Temmerman S, D'Alpaos A, et al Numerical models of salt marsh evolution: Ecological, geomorphic, and climatic factors. Reviews of Geophysics. 2012;50(1):RG1002.

[58] Passeri DL, Hagen SC, Medeiros SC, Bilskie MV, Alizad K, Wang D. The dynamic effects of sea level rise on low-gradient coastal landscapes: A review. Earth's Future. 2015;3(6):159–181.

[59] Alizad K, Hagen SC, Morris JT, Medeiros SC, Bilskie MV, Weishampel JF. Coastal wetland response to sea-level rise in a fluvial estuarine system. Earth's Future. 2016;4:483–497.

[60] Alizad K, Hagen SC, Morris JT, Bacopoulos P, Bilskie MV, Weishampel JF, et al A coupled, two-dimensional hydrodynamic-marsh model with biological feedback. Ecological Modelling. 2016;327:29–43.

[61] Mendelssohn IA, Seneca ED. The influence of soil drainage on the growth of salt marsh cordgrass *Spartina alterniflora* in North Carolina. Estuarine and Coastal Marine Science. 1980;11:27–40.

[62] Steyer GD, Sasser CE, Visser JM, Swenson EM, Nyman JA, Raynie RC. A proposed coast-wide reference monitoring system for evaluating wetland restoration trajectories in Louisiana. Environ Monit Assess. 2003;81:107–117. 12620009

[63] Ross MS, Ruiz PL, Sah JP, Hanan EJ. Chilling damage in a changing climate in coastal landscapes of the subtropical zone: a case study from south Florida. Global Change Biology. 2009;15(7):1817–1832.

[64] Osland MJ, Day RH, Hall CT, Brumfield MD, Dugas JL, Jones WR. Mangrove expansion and contraction at a poleward range limit: climate extremes and land-ocean temperature gradients. Ecology. 2017;98:125–137. doi: 10.1002/ecy.1625 27935029 10.1002/ecy.1625

[65] Osland MJ, Day RH, From AS, McCoy ML, McLeod JL, Kelleway JJ. Life stage influences the resistance and resilience of black mangrove forests to winter climate extremes. Ecosphere. 2015;6:art160.

[66] Cavanaugh KC, Kellner JR, Forde AJ, Gruner DS, Parker JD, Rodriguez W, et al Poleward expansion of mangroves is a threshold response to decreased frequency of extreme cold events. Proceedings of the National Academy of Sciences. 2014;111:723–727. 10.1073/pnas.1315800111 PMC3896164 24379379

[67] Woodroffe CD, Grindrod J. Mangrove biogeography: The role of quaternary environmental and sea-level change. Journal of Biogeography. 1991;18:479–492.

[68] Cavanaugh KC, Parker JD, Cook-Patton SC, Feller IC, Williams AP, Kellner JR. Integrating physiological threshold experiments with climate modeling to project mangrove species’ range expansion. Global Change Biology. 2015;21:1928–1938. doi: 10.1111/gcb.12843 25558057 10.1111/gcb.12843

[69] Withers K. Wind-tidal flats In: Tunnell JW, Judd FW, editors. The Laguna Madre of Texas and Tamaulipas. College Station, Texas, USA: Texas A&M University Press; 2002 p. 114–126.

[70] Longley WL. Estuaries In: North GR, Schmandt J, Clarkson J, editors. The Impact of Global Warming on Texas: A Report to the Task Force on Climate Change in Texas. Austin, TX, USA: The University of Texas; 1995 p. 88–118.

[71] Montagna PA, Gibeaut JC, Tunnell JW Jr. South Texas climate 2100: coastal impacts In: Norwine J, John K, editors. The Changing Climate of South Texas 1900–2100: Problems and Prospects, Impacts and Implications. Kingsville, Texas, USA: CREST-RESSACA. Texas A & M University; 2007 p. 57–77.

[72] Bucher D, Saenger P. A classification of tropical and subtropical Australian estuaries. Aquatic Conservation: Marine and Freshwater Ecosystems. 1994;4(1):1–19.

[73] Deegan LA, Day JW Jr., Gosselink JG, Yañez-Arancibia A, Soberón-Chàvez G, Sànchez-Gil P. Relationships among physical characteristics, vegetation distribution and fisheries yield in Gulf of Mexico estuaries In: Wolfe DA, editor. Estuarine Variability. Orlando, Florida USA: Academic Press; 1986 p. 83–100.

[74] Lovelock CE, Feller IC, Reef R, Hickey S, Ball MC. Mangrove dieback during fluctuating sea levels. Scientific Reports. 2017;7(1):1680 doi: 10.1038/s41598-017-01927-6 28490782 10.1038/s41598-017-01927-6 PMC5431776

[75] Duke NC, Kovacs JM, Griffiths AD, Preece L, Hill DJE, van Oosterzee P, et al Large-scale dieback of mangroves in Australia’s Gulf of Carpentaria: a severe ecosystem response, coincidental with an unusually extreme weather event. Mar Freshw Res. 2017; doi: 10.1071/MF16322

[76] Diop ES, Soumare A, Diallo N, Guisse A. Recent changes of the mangroves of the Saloum River Estuary, Senegal. Mangroves and Salt Marshes. 1997;1:163–172.

[77] Eslami-Andargoli L, Dale P, Sipe N, Chaseling J. Mangrove expansion and rainfall patterns in Moreton Bay, southeast Queensland, Australia. Estuarine, Coastal and Shelf Science. 2009;85(2):292–298.

[78] Asbridge E, Lucas R, Accad A, Dowling R. Mangrove response to environmental changes predicted under varying climates: case studies from Australia. Current Forestry Reports. 2015;1(3):178–194.

[79] Rasser MK, Fowler NL, Dunton KH. Elevation and plant community distribution in a microtidal salt marsh of the western Gulf of Mexico. Wetlands. 2013;33:575–583.

[80] Eleuterius LN, Eleuterius CK. Tide levels and salt marsh zonation. Bulletin of Marine Science. 1979;29(3):394–400.

[81] Odum WE, McIvor CC, Smith III TJ. The ecology of mangroves of south Florida: a community profile: U.S. Fish and Wildlife Service, Office of Biological Services, Washington, D.C. FWS/OBS-81/24; 1982.

[82] Lugo AE, Snedaker SC. The ecology of mangroves. a. 1974;5(1):39–64.

[83] Twilley RR, Day JW. Mangrove wetlands In: Day JW, Crump BC, Kemp MW, Yáñez-Arancibia A, editors. Estuarine Ecology. Hoboken, New Jersey, USA: John Wiley & Sons; 2012 p. 165–202.

[84] Ibáñez C, Morris JT, Mendelssohn IA, Day JW. Coastal marshes In: Day JW, Crump BC, Kemp MW, Yáñez-Arancibia A, editors. Estuarine Ecology, Second Edition Hoboken, New Jersey, USA: John Wyley and Sons; 2012 p. 129–163.

[85] Day JW, Boesch DF, Clairain EJ, Kemp GP, Laska SB, Mitsch WJ, et al Restoration of the Mississippi Delta: lessons from hurricanes Katrina and Rita. Science. 2007;315(5819):1679–1684. doi: 10.1126/science.1137030 17379799 10.1126/science.1137030

[86] Twilley RR, Bentley SJ, Chen Q, Edmonds DA, Hagen SC, Lam NS-N, et al Co-evolution of wetland landscapes, flooding, and human settlement in the Mississippi River Delta Plain. Sustainability Science. 2016;11:711–731. 10.1007/s11625-016-0374-4 PMC6106107 30174740

[87] Blum MD, Roberts HH. Drowning of the Mississippi Delta due to insufficient sediment supply and global sea-level rise. Nature Geoscience. 2009;2(7):488–491.

[88] Couvillion BR, Barras JA, Steyer GD, Sleavin W, Fischer M, Beck H, et al. Land area change in coastal Louisiana from 1932 to 2010: U.S. Geological Survey Scientific Investigations Map 3164, scale 1:265,000, 12 p. pamphlet. 2011.

[89] Kintisch E. Can coastal marshes rise above it all? 2013;341:480–481. 10.1126/science.341.6145.480 23908219

[90] Couvillion BR, Steyer GD, Wang H, Beck HJ, Rybczyk JM. Forecasting the effects of coastal protection and restoration projects on wetland morphology in coastal Louisiana under multiple environmental uncertainty scenarios. Journal of Coastal Research. 2013;67(sp1):29–50.

[91] Whelan KRT, Smith TJ III, Anderson GH, Ouellette ML. Hurricane Wilma’s impact on overall soil elevation and zones within the soil profile in a mangrove forest. Wetlands. 2009;29(1):16–23.

[92] Cahoon DR, Lynch JC. Vertical accretion and shallow subsidence in a mangrove forest of southwestern Florida, U.S.A. Mangroves and Salt Marshes. 1997;1:173–186.

[93] Smith TJ III, Anderson GH, Balentine K, Tiling G, Ward GA, Whelan KR. Cumulative impacts of hurricanes on Florida mangrove ecosystems: sediment deposition, storm surges and vegetation. Wetlands. 2009;29(1):24–34.

[94] Smith III TJ. Development of a long-term sampling network to monitor restoration success in the southwest coastal Everglades: vegetation, hydrology, and sediments. U.S. Geological Survey, Fact Sheet FS-2004-30152004.

[95] Morris JT. Estimating net primary production of salt marsh macrophytes In: Fahey TJ, Knapp AK, editors. Principles and Standards for Measuring Primary Production. New York, New York, USA: Oxford University Press; 2007 p. 106–119.

[96] Morris JT, Sundberg K, Hopkinson CS. Salt marsh primary production and its responses to relative sea level and nutrients in estuaries at Plum Island, Massachusetts, and North Inlet, South Carolina, USA. Oceanography. 2013;26(3):78–84.

[97] Kirwan ML, Guntenspergen GR. Response of plant productivity to experimental flooding in a stable and a submerging marsh. Ecosystems. 2015;18(5):903–913.

[98] Snedden GA, Cretini K, Patton B. Inundation and salinity impacts to above-and belowground productivity in Spartina patens and Spartina alterniflora in the Mississippi River Deltaic Plain: implications for using river diversions as restoration tools. Ecological Engineering. 2015;81:133–139.

[99] Langley AJ, Mozdzer TJ, Shepard KA, Hagerty SB, Megonigal PJ. Tidal marsh plant responses to elevated CO_2_, nitrogen fertilization, and sea level rise. Global Change Biology. 2013;19(5):1495–1503. doi: 10.1111/gcb.12147 23504873 10.1111/gcb.12147

[100] Schile LM, Callaway JC, Suding KN, Kelly NM. Can community structure track sea-level rise? Stress and competitive controls in tidal wetlands. Ecology and Evolution. 2017;7(4):1276–1285. doi: 10.1002/ece3.2758 28303196 10.1002/ece3.2758 PMC5305999

[101] Morris JT. Ecological engineering in intertidial saltmarshes. Hydrobiologia. 2007;577(1):161–168.

[102] Cherry JA, Ramseur GS, Sparks EL, Cebrian J. Testing sea-level rise impacts in tidal wetlands: a novel *in situ* approach. Methods in Ecology and Evolution. 2015;6(12):1443–1451.

[103] Stagg CL, Mendelssohn IA. Controls on resilience and stability in a sediment-subsidized salt marsh. Ecological Applications. 2011;21(5):1731–1744. 21830714 10.1890/09-2128.1

[104] Krauss KW, Cormier N, Osland MJ, Kirwan ML, Stagg CL, Nestlerode JA, et al Created mangrove wetlands store belowground carbon and surface elevation change enables them to adjust to sea-level rise. Scientific Reports. 2017;7:1030 doi: 10.1038/s41598-017-01224-2 28432292 10.1038/s41598-017-01224-2 PMC5430729

[105] Callaway JC, DeLaune RD, Patrick WH. Sediment accretion rates from four coastal wetlands along the Gulf of Mexico. Journal of Coastal Research. 1997;13:181–191.

[106] DeLaune RD, Patrick WH, Buresh RJ. Sedimentation rates determined by 137Cs dating in a rapidly accreting salt marsh. Nature. 1978;275(5680):532–533.

[107] Turner RE, Milan CS, Swenson EM. Recent volumetric changes in salt marsh soils. Estuarine, Coastal and Shelf Science. 2006;69(3):352–359.

[108] Lynch JC, Meriwether JR, McKee BA, Vera-Herrera F, Twilley RR. Recent accretion in mangrove ecosystems based on 137 Cs and 210 Pb. Estuaries and Coasts. 1989;12(4):284–299.

[109] Nyman JA, DeLaune RD, Patrick WH. Wetland soil formation in the rapidly subsiding Mississippi River Deltaic Plain: Mineral and Organic matter relationships. Estuarine, Coastal and Shelf Sciences. 1990;31:57–69.

[110] Scholl DW. Recent sedimentary record in mangrove swamps and rise in sea level over the sourthwestern coast of Florida Part 1. Marine Geology. 1964;1:344–366.

[111] Breithaupt JL, Smoak JM, Smith TJ III, Sanders CJ. Temporal variability of carbon and nutrient burial, sediment accretion, and mass accumulation over the past century in a carbonate platform mangrove forest of the Florida Everglades. Journal of Geophysical Research: Biogeosciences. 2014;119(10):2032–2048.

[112] Comeaux RS, Allison MA, Bianchi TS. Mangrove expansion in the Gulf of Mexico with climate change: Implications for wetland health and resistance to rising sea levels. Estuarine, Coastal and Shelf Science. 2012;96:81–95.

[113] Parkinson RW, DeLaune RD, White JR. Holocene sea-level rise and the fate of mangrove forests within the wider caribbean region. Journal of Coastal Research. 1994;10(4):1077–1086.

[114] Morris JT, Barber DC, Callaway JC, Chambers R, Hagen SC, Hopkinson CS, et al Contributions of organic and inorganic matter to sediment volume and accretion in tidal wetlands at steady state. Earth's future. 2016;4(4):110–121. doi: 10.1002/2015EF000334 27819012 10.1002/2015EF000334 PMC5074445

